# Supplementary material for: Evidence supported by Mendelian randomization: impact on inflammatory factors in knee osteoarthritis
Source: Front Med (Lausanne). 2024 May 28;11:1382836. doi: 10.3389/fmed.2024.1382836 (PMC11165061; doi:10.3389/fmed.2024.1382836)
Supplement: Supplementary file 1 [file Data_Sheet_1.ZIP › data/GCST90274843_5e6/843.forest.pdf]

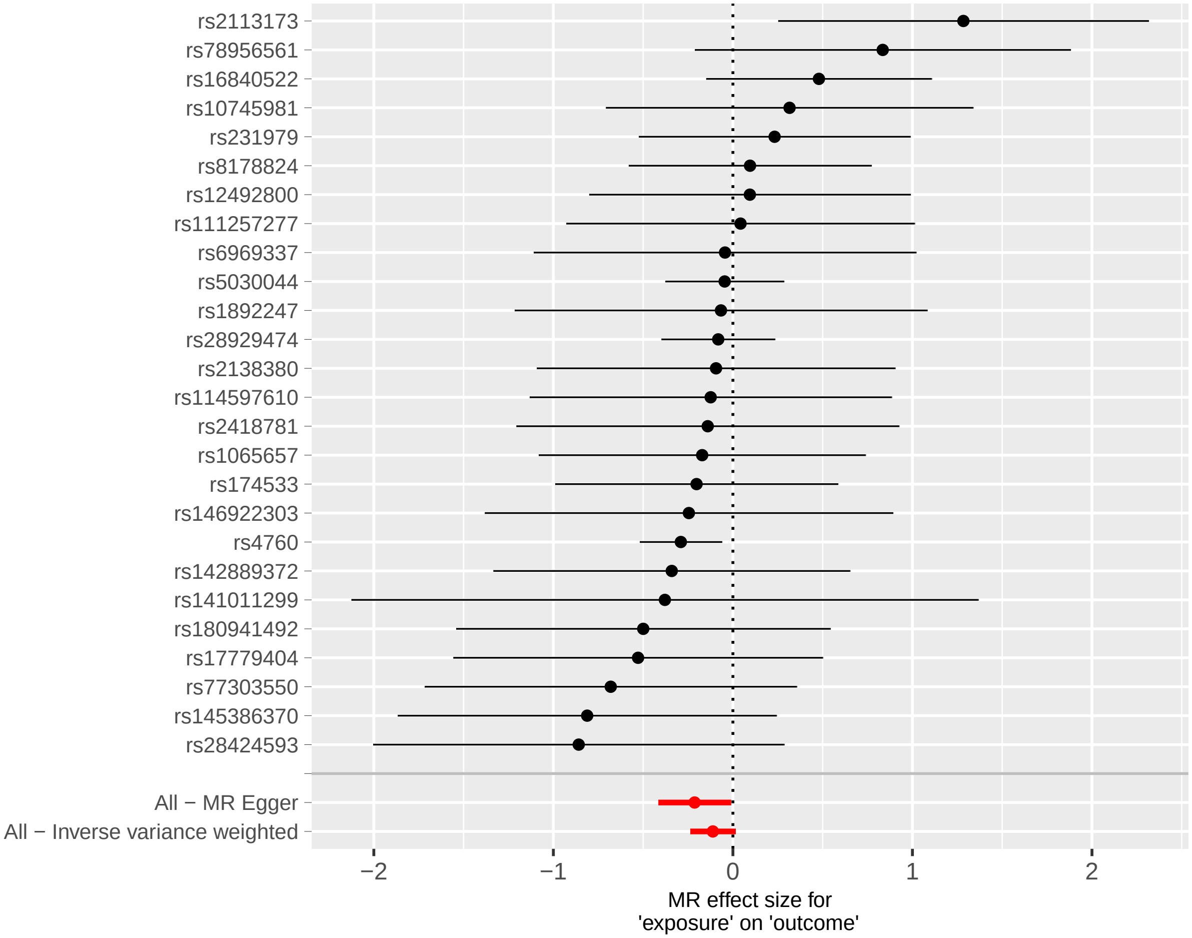
rs2113173
rs78956561
rs16840522
rs10745981
rs231979
rs8178824
rs12492800
rs111257277
rs6969337
rs5030044
rs1892247
rs28929474
rs2138380
rs114597610
rs2418781
rs1065657
rs174533
rs146922303
rs4760
rs142889372
rs141011299
rs180941492
rs17779404
rs77303550
rs145386370
rs28424593
All − MR Egger
All − Inverse variance weighted
−2
−1
0
1
2
MR effect size for
'exposure' on 'outcome'
